# Decapitation in Rats: Latency to Unconsciousness and the ‘Wave of Death’

**DOI:** 10.1371/journal.pone.0016514

**Published:** 2011-01-27

**Authors:** Clementina M. van. Rijn, Hans Krijnen, Saskia Menting-Hermeling, Anton M. L. Coenen

**Affiliations:** Department of Biological Psychology, Donders Institute for Brain, Cognition and Behaviour, Radboud University Nijmegen, Nijmegen, The Netherlands; Université Pierre et Marie Curie, France

## Abstract

The question whether decapitation is a humane method of euthanasia in awake animals is being debated. To gather arguments in this debate, obsolete rats were decapitated while recording the EEG, both of awake rats and of anesthetized rats. Following decapitation a fast and global loss of power of the EEG was observed; the power in the 13–100 Hz frequency band, expressing cognitive activity, decreased according to an exponential decay function to half the initial value within 4 seconds. Whereas the pre-decapitation EEG of the anesthetized animals showed a burst suppression pattern quite different from the awake animals, the power in the postdecapitation EEG did not differ between the two groups. This might indicate that either the power of the EEG does not correlate well with consciousness or that consciousness is briefly regained in the anesthetized group after decapitation. Remarkably, after 50 seconds (awake group) or 80 seconds (anesthetized group) following decapitation, a high amplitude slow wave was observed. The EEG before this wave had more power than the signal after the wave. This wave might be due to a simultaneous massive loss of membrane potentials of the neurons. Still functioning ion channels, which keep the membrane potential intact before the wave, might explain the observed power difference. Two conclusions were drawn from this experiment. It is likely that consciousness vanishes within seconds after decapitation, implying that decapitation is a quick and not an inhumane method of euthanasia. It seems that the massive wave which can be recorded approximately one minute after decapitation reflects the ultimate border between life and death. This observation might have implications in the discussions on the appropriate time for organ donation.

## Introduction

Decapitation is a procedure to euthanize small animals, such as rats and birds. It is achieved by swiftly cutting the neck of the animal close to the head, by using a guillotine with a sharp blade. The advantage of this technique is that it provides a means to obtain brain tissues and fluids that are not contaminated with chemicals such as gases and anesthetics, and not affected by electrical currents. However, the method is not free from controversies and still not generally accepted as an acceptable humane euthanasia procedure [1]. This is so although the majority of researchers conclude that decapitation is not an inhumane technique [2], [3], [4]. A minority of authors, however, argue that decapitation may induce distress and pain to the animals [5]. The controversy is mainly created by the uncertain interpretation of the brain activity seen from the moment of the neck cut to the point that the EEG is iso-electric, indicating deep unconsciousness. The duration to EEG iso-electricity varies from a few seconds [3] to 14 seconds [5], this discrepancy is considerable. Given the debate about this matter, there is no consensus about the acceptance of decapitation as an adequate euthanasia method in the Animal Ethical Committees in The Netherlands, having the task to consider whether animal experiments are allowed. The question how long consciousness is present after a neck cut is essential to gauge the humane nature and acceptability of decapitation.

The present experiment was undertaken in order to analyze in detail the EEG characteristics following decapitation in rats. A group of awake, conscious rats was decapitated while the EEG was recorded before, during and after the severing of the neck. In order to facilitate the interpretation of the EEG in terms of consciousness, a second group of rats were decapitated under anesthesia. Before decapitation these rats were anesthetized to uncover which kind of activity can be present in the EEG, not indicating a form of consciousness. This conscious-anesthesia design allowed a comparison of EEG characteristics of conscious and unconscious rats, in particular with respect to the critical period between the moment of decapitation and the complete disappearance of EEG activity.

## Methods

Permission for the experiment was obtained from the Ethical Animal Committee of the Radboud University Nijmegen under number RU-DEC 2007-170. Twenty-two male Wistar rats of 6 to 8 months old with weights between 230 and 290 grams, coming from a previous electrophysiological experiment (RU DEC 2007-034), served as experimental subjects. All were already provided with a permanent tripolar electrode set. Two electrodes were aimed at the frontal and parietal cortex, the ground electrode was placed above the cerebellum.

Decapitation took place under two experimental conditions. In the first condition (n = 9) rats were fully conscious, while in the second condition (n = 8) rats were under isoflurane anesthesia, in a dose that is commonly used for operation purposes. The experiment started with 22 rats, but 5 rats lost the electrode set during the experiment.

The rats were immobilized by an experienced animal technician, put with their head in the guillotine opening and an EEG cable was connected. A baseline EEG was recorded for 30 seconds. Subsequently the animals were decapitated swiftly. The guillotine was constructed by the Mechanical Workshop of the Faculty of Social Sciences. It consisted of a metal frame and a sharp blade, which could be operated by one hand. During decapitation the EEG was recorded while the animals were observed. EEG recordings were continued for at least 5 minutes after decapitation.

EEGs were registered in the frequency band from 0.1–100 Hz, with a notch filter of 50 Hz, digitized with a sample frequency of 512 Hz using a Windaq system (www.dataq.com), and stored for off-line analysis.

Following decapitation all rats were anatomically inspected to determine the exact plane of section. This section was aimed at the atlanto-occipital joint to block as much sensory input to the brain as possible [2]. All animals, except three, had a correct spinal section. In these three animals the section had a distance of more than one vertebra to the atlanto-occipital joint. However, data from these rats could not be distinguished from those with a correct section and were pooled to the correctly decapitated animals.

The analysis of the EEG was performed using Brain Vision Analyzer (www.brainproducts.com). EEG traces were segmented in epochs of 1.0 seconds with a sliding window of 0.5 seconds. On these epochs FFT was performed with a low cutoff of 1.0 Hz and a high cutoff of 100 Hz, both with 48 dB/oct. The one second epochs just before and after the decapitation artifact were ignored. The output of the FFT was voltage density (V/Hz), which is referred to as the power of the EEG. GraphPad Prism 5.03 (www.graphpad.com) was used for the determination of the time course of the power of the EEG. Statistical analysis was performed in SSPS 15.0 (www.spss.com).

## Results

**Figure 1:**
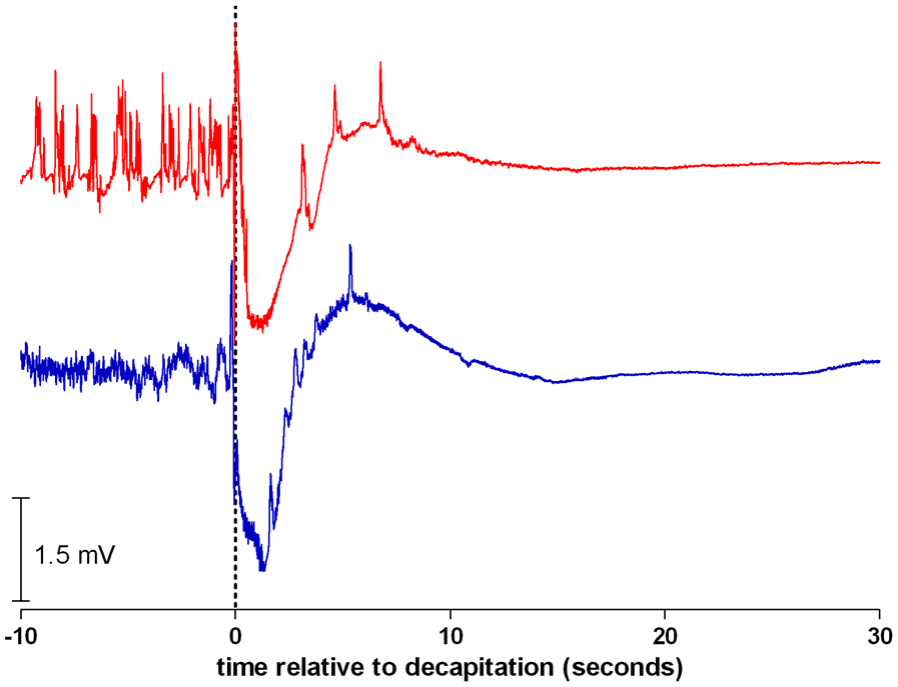
EEGs of rats after decapitation. Examples of EEG of rats recorded from 10 seconds before until 30 seconds after decapitation. Band pass 0.1–100 Hz. Lower trace of an awake rat, upper trace of an anesthetized rat. Note the large artifact of the guillotine and the artifacts coinciding with chewing movements of the mouth.

The lower trace of Figure 1 shows an example of an EEG of a rat of the awake group recorded just before (i.e. baseline), during and after decapitation. The baseline EEGs of these rats are fully representative for alert, awake animals. After decapitation, a low amplitude EEG is seen, superimposed on a large wave, presumably an artifact of the guillotine blade. In all animals this low amplitude EEG activity is interlarded with repetitive smaller artifacts, which coincided with chewing movements of the animal's mouth. These movements have a frequency of about 1 Hz and last for about 15 seconds. In observing the headless body of the rats, a repetitive synchronous jerking of the hind legs is visible, lasting for about one minute.

The upper trace of Figure 1 shows the EEG of a rat of the anesthetized group. The baseline EEG of these rats shows a typical burst-suppression anesthetic pattern. After the guillotine artifact the chewing movements are also observable in these EEGs. Also jerking of the legs is observed.

**Figure 2:**
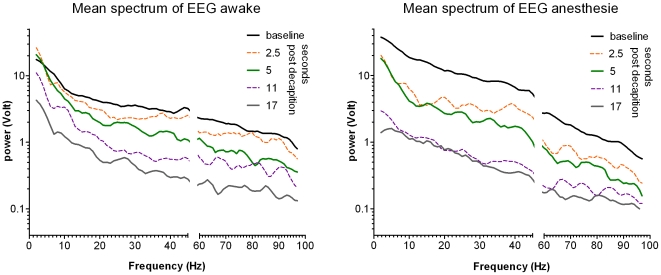
Spectrograms of the EEG after decapitation. Mean spectrograms of the EEGs for the baseline period, i.e. the 10 seconds before decapitation, and for 5 time points post decapitation. Data are in Volt, group means are given. On the raw mean spectrum 2nd order smoothing with 4 neighbors was applied. On the abscissa the data around 50 Hz are deleted due to the notch filter. Please note the log scale of the ordinate. In anesthesia, before decapitation the EEG has high power content, reflecting the bursts in the burst suppression pattern. Following decapitation in both groups, a fast and global decay of the power is observed. The quantification of this decay is shown in fig. 3.

**Figure 3:**
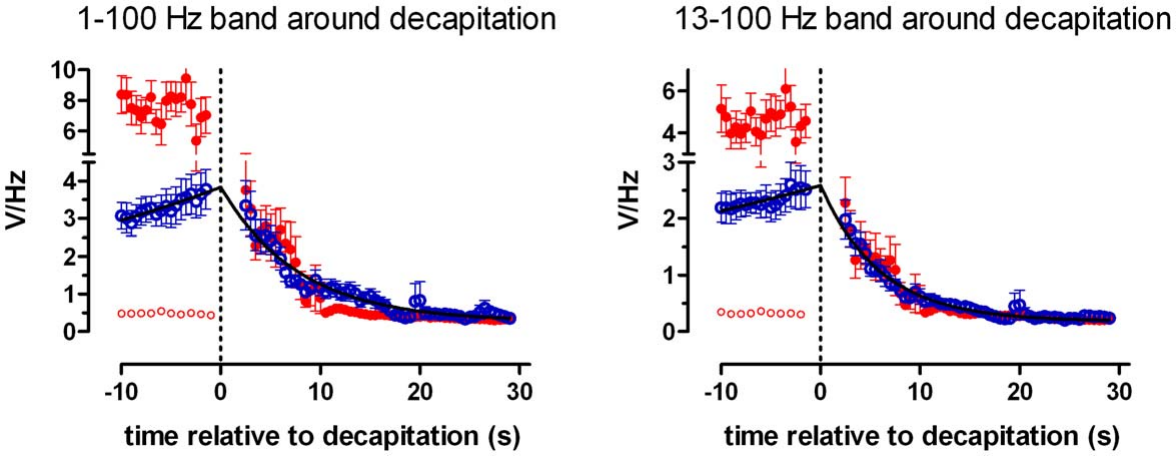
Time dependency of the power of the EEG after decapitation. Time dependency of the mean power of the EEG between 1 and 100 Hz (al) and between 13–100 Hz (b). Blue open symbols for the awake group, red symbols for the anesthetized group. In the pre-decapitation EEG of the awake animals the power increases with 30% during the last 10 seconds. The power of the pre-decapitation period for the anesthetized group is given for the burst (red closed symbols, high power) as well as for the suppression parts (red open symbols, low power). The power of all rats sharply decreases after decapitation. An exponential decay function described this time dependent decrease. For both groups the half time was 5.5 seconds for the 1–100 Hz band and 3.7 seconds for the 13–100 Hz band.

FFT of the EEG signals was performed. Figure 2 shows the averaged spectrograms of the baseline periods and of 4 time points after decapitation (2.5; 5; 11 and 17 seconds). A time dependent decrease of the power is observed. The quantification of this time dependency is shown in Figure 3.

Figure 3a shows the time dependency of the mean power of the EEG between 1 and 100 Hz of all rats of both groups (45–55 Hz was excluded because of the notch filter). In the baseline EEG of the awake animals, the power increases with 30% during the last 10 seconds before decapitation, reaching 4.0 (0.3) V/Hz (fitted value with SE of fit). In the anesthetized group the power of the baseline period is estimated for the burst as well as for the suppression parts. The suppression part has a very low power, while the power during bursts is, as expected, quite high.

The EEG power of all rats sharply decreases after decapitation. An exponential decay function was fitted to these post-decapitation data, with a start value of the fitted pre-decapitation value. For the data of the awake group and the anesthetized group, independent fits were compared to a global fit that shared the rate of decay. The independent fits did not differ significantly (p = 0.5), therefore the preferred model was the global fit: in 5.5 seconds the power reduced to 50% of the start value level, with a 95% confidence interval between 5.0 and 6.1 s. The power of the post-decapitation EEG in the anesthetized group is thus closely the same as in the awake group.

Figure 3b shows the power of the EEG in the band, between 13 and 100 Hz. The power in this band is recognized as expressing vigilance and the ability to experience sensory perceptions, including pain, whereas the lack of activity in this band is interpreted as an unequivocal loss of sensibility [6], [7], [8]. The power of the post decapitation EEG in this cognitive band has a time course comparable with the power in the 1–100 Hz band, but the time to half-life is shorter: 3.7 seconds, with a 95% confidence interval between 3.5 to 4.1 seconds (significantly different from the previous 5.5 seconds, t(2)  = 173, p<0.001).

**Figure 4:**
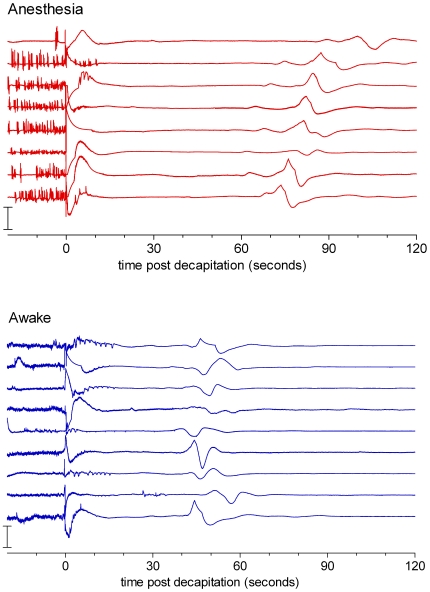
All EEGs until 120 seconds after decapitation. All EEGs recorded from 10 seconds before until 120 seconds after decapitation. Band pass 0.1–100 Hz. Lower panel awake animals, upper panel anesthetized group. Note the large slow wave around 50 seconds after decapitation for the awake animals and around 80 seconds fir the anesthetized group.

Figure 4 shows long EEG tracings of all the animals of the experiment. A relatively long time after decapitation, when the EEG has amply reached iso-electricity, defined as at most 10% power left, a large amplitude slow positive-negative-positive wave is observed in all rats. The latency of this wave after decapitation in the awake group is 52±7 seconds, in the anesthetized group 85±6 seconds.

**Figure 5:**
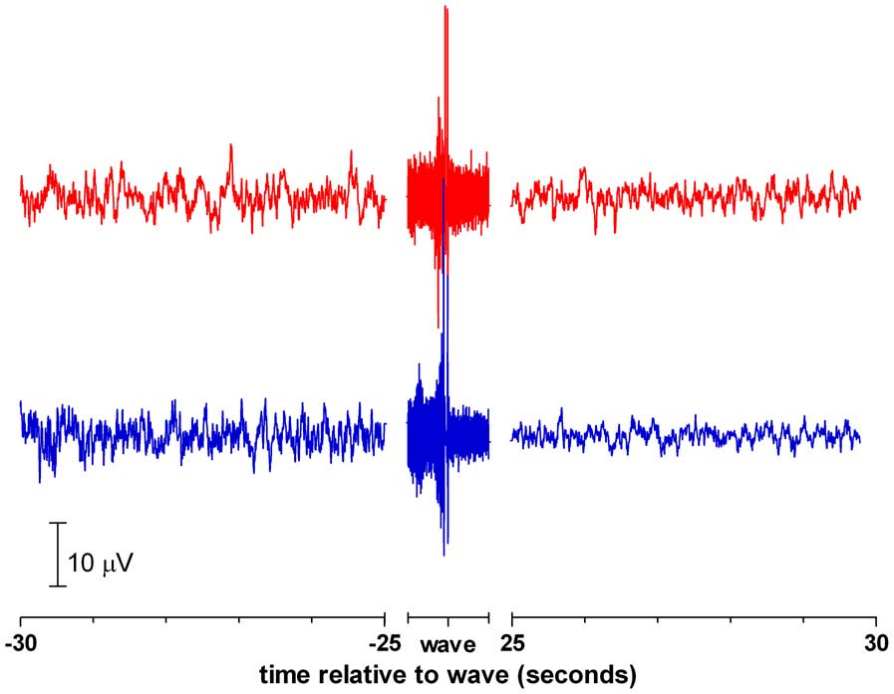
EEGs around the wave. Examples of EEG of rats recorded from 30 seconds before the wave until 30 seconds after the wave. Band pass 0.1–100 Hz. Lower trace of an awake rat, upper trace of an anesthetized rat. Note the scale of the ordinate. This EEG is assumed to be iso-electric, but the power after the wave is lower then that before the wave.

**Figure 6:**
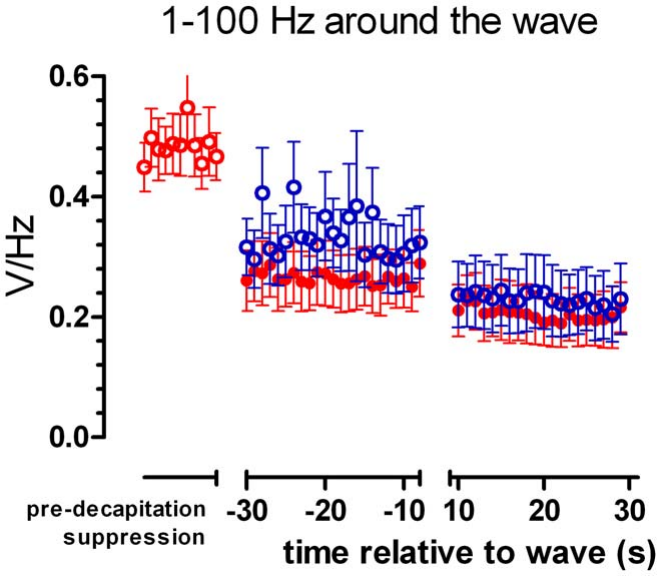
Power of the EEGs around the wave. The mean power of the EEG between 1 and 100 Hz around the wave. Blue open symbols for the awake group, red closed symbols for the anesthetized group. In the post-wave EEG the power is lower then that in the pre-wave EEG.

Although the pre-wave as well as the post-wave EEG is iso-electric, visually and according to the 10% definition, an enlargement of the EEG trace just before and after this wave shows a difference in EEG amplitude (see Figure 5). FFT analysis (Figure 6) showed indeed a significant difference in power in all frequency bands. The 1–100 band is shown: in a two way ANOVA, merging 20 one-second epochs, with group as between factor and pre-post wave as within factor, a highly significant pre-post wave difference was found F(1,15)  = 42, p<0.0001).

## Discussion

The critical point for judging decapitation as a humane technique for euthanizing animals is the time it takes after decapitation for animals to become fully unconscious, unable to perceive distress and pain. The aim of this study was to find an answer in the EEG.

In awake rats it lasts takes about 17 seconds before the power of the EEG is iso-electric. Since it is not known how the power of the EEG correlates with the level of consciousness, in drug free subjects, iso-electricity forms a solid base to regard the animal as completely unconscious. However, the question arises whether the degree of consciousness at an earlier time is already so low that perception of pain and distress is already totally eliminated.

In the literature, it is not easy to find data on the relation EEG power -consciousness in unmedicated individuals. Reports on this relation are either in damaged brains, such as coma patients [9], or during pharmacological induced unconsciousness, such as anesthesia [10]. Therefore, for the present discussion, EEGs were taken from our database, of waking and sleeping rats and the power of these EEGs were compared, to get an indication how the power of a sleep EEG, when consciousness is nearly absent, is related to the waking EEG, when consciousness is high. It is well known that the shape of the spectrograms differ between these two states [11]; the power in the low frequencies (1–12 Hz) during sleep is much higher then that of the awake state (200%, SEM 21%, n = 8 rats, 10 seconds per animal). For the present question is relevant that the power in the cognitive band (13–100 Hz) of the sleep state is still 78%, (SEM 4.4%) of that during waking. Since sleep is not deeply unconscious, it seems save to take a lower value, thus to assume that the animals are unconscious at a power decrease of the cognitive band of 50%. This point is reached in 3.7 seconds.

The EEG of an anesthetized subject is very different from that of an unmedicated state. Therefore it is quite surprising that the post-decapitation EEG, and its power, of the anesthetized group are almost the same as that of the awake group. One interpretation is that the resulting power of the EEG in the awake animals is not well indicative of consciousness and distress, since the same activity is present in the EEG of non-conscious anesthetized animals. On the other hand, the resemblance of the power of the post decapitation EEG in both groups might also imply that the animal's consciousness is briefly enhanced immediately after the neck cut. The interpretation might be that the cut is such a powerful arousal stimulus, that even anesthetized animals regain consciousness. This statement is supported by the observation that the correlation dimension of the EEG, a measure for consciousness, sharply rises when a painful stimulus is given to an animal in deep anesthesia [12].

The results presented here warrant the conclusion that decapitation leads to a rapid loss of consciousness, so it seems safe to assume that in 3–4 seconds after decapitation the animal is unconscious, unable to perceive stress and pain. This number corresponds well with results of Derr [3]: 2.7 seconds and of Holson [4]; 3–6 seconds. It might be concluded that decapitation is not an inhumane method for euthanizing small animals. This conclusion, however, does not imply that decapitation is recommended in all situations. It is an offensive method which has an esthetical disadvantage: performing and observing this technique is displeasing. Moreover, achieving decapitation requires experienced and skilled personnel.

A remarkable and consistent finding is the occurrence of a very slow, large, late wave in the EEG. A relatively long time after cutting the neck, when iso-electricity is already present in the EEG for a considerable time, a large amplitude positive-negative-positive wave follows at approximately 50 seconds after decapitation of rats of the awake group. In the anesthetized rats this wave is also present but comes later, at about 80 seconds after the neck cut. By carefully inspecting the EEG trace just before and after this wave, it could be seen the amplitude of the EEG is higher that before the wave than after the wave; this was confirmed by FFT analysis. The positive-negative-positive shape of the wave is likely to be brought about by the high-pass filter of 0.1 Hz. It is possible that the original wave is a sigmoid shaped wave, as described by Bureš and Burešová [13]. It is speculated here that, due to lack of energy to maintain this potential, neurons lose their membrane potential at this time. The wave thus might reflect a massive opening of ion channels: a depolarization wave. The still functioning of these ion channels in the period before the wave might be responsible for the observed difference in the pre-wave and post-wave power of the EEG. Hence, it is thought that the wave represents the synchronous death of brain neurons, expressed in a ‘wave of death’.

**Figure 7:**
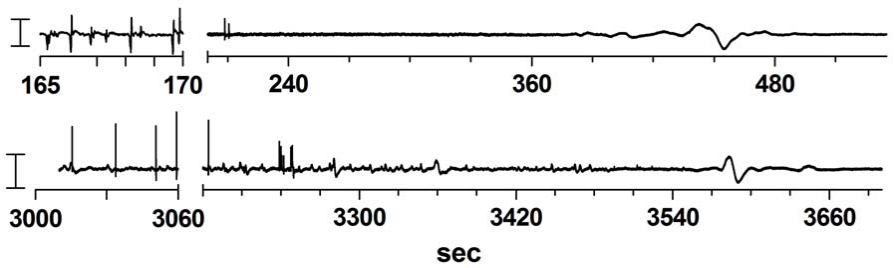
EEG of two animals euthanatized with an overdose of pentobarbital. EEG of two animals euthanatized with an overdose of pentobarbital. The time after injection of 120 mg/kg is indicated. The EEG before the break of the abscissa is a typical burst suppression pattern A considerable time after the last burst, a large wave is observed. Note the large difference in the time-delay after injection and after the last burst.

In the anesthetized group this wave is delayed, perhaps because of the protective character of the anesthetic drugs. Also in the EEG of rats euthanized by an overdose of pentobarbital such a wave can be observed (see figure 7 for two examples). In a recent paper by Lakhmir and colleagues [14] the EEG of patients was recorded in the form of a bispectral index monitor (BIS) during their dying. In all patients they could record a large wave, near death, which they interpreted as related to the loss of cellular membrane potentials. Perhaps, this wave has some analogies with the ‘wave of death’, although with frequency characteristics are different from the wave described in the present experiment.

It is concluded from this experimental work that consciousness is likely to vanish within some seconds after decapitation. It is therefore implied that decapitation is a quick and not inhumane method of euthanasia. Interestingly, it seems that it takes nearly one minute for neurons to loose their membrane potentials. In view of this finding, a long EEG monitoring during the process of natural dying and basic research regarding the physiology of brain functioning during this process might give information in the ongoing discussion on the definition of brain death, but it is suggested here that the massive wave which can recorded approximately one minute after decapitation ultimately reflects brain death.
